# Detection of human papillomavirus-16 in ovarian malignancy

**DOI:** 10.1038/sj.bjc.6601172

**Published:** 2003-08-12

**Authors:** Q-J Wu, M Guo, Z-M Lu, T Li, H-Z Qiao, Y Ke

**Affiliations:** 1 Inner Mongolia Medical College, Hohehot Municipality 010000, China; 2 Laboratory of Genetics, Beijing Institute for Cancer Research, School of Oncology, Peking University, No.1 Da-Hong-Luo-Chang St. West District, Beijing 100034, China

**Keywords:** human papillomavirus-16, p53 tumour suppressor gene, ovarian malignancy

## Abstract

Human papillomavirus is the causal factor for cervical cancer. However, the role of HPV infection in ovarian cancer is unclear. This study aimed to determine the presence of human papillomavirus-16 (HPV-16) in ovarian cancer tissues. Archived human ovarian cancer tissues (*N*=54 cases, 50 are epithelial cancer, four are nonepithelial cancer) embedded in paraffin blocks were used. Controls are 30 nonmalignant ovarian tissue blocks. *In situ* hybridisation (ISH) and immunohistochemistry (IHC) were used to detect the presence of HPV-16 and p53 expression. In all, 52 or 36% of the epithelial ovarian tumours detected by ISH or IHC, respectively, were HPV-16 E6 positive. In contrast, only 6.7% of normal ovarian tissues were HPV-16 positive proved by ISH. Human papillomavirus-16 infection was significantly higher in cancer tissues compared to controls with an odds ratio of 16.7 (95% confidence interval [CI]=3.2–71.4, *P*<0.01). No significant correlation between HPV-16 infection and histological types of cancer was found (*P*>0.05). p53 gene expression was detected in 42% epithelial ovarian cancers. No correlation between p53 expression and HPV-16 infection was found. The results showed the presence of HPV-16 E6 in ovarian carcinoma, suggesting that HPV infection might play a role in ovarian carcinogenesis.

Human papillomavirus (HPV) infection has been identified as the necessary cause of cervical cancer (Bosch *et al*, 2002). The progression of proliferative epithelial cells at different body sites to carcinoma may also be associated with the high-risk type HPV infection (zur Hausen, 1999). Ovarian tumour is a common neoplasm of the female genital tract and one of the most lethal gynaecologic malignancies (Prazzini *et al*, 1997). The aetiology of ovarian cancer remains unclear (Boyle *et al*, 2000). Malignancy of epithelial origin accounts for 85–90% of the total ovarian tumour morbidity. Therefore, the involvement of HPV infection in epithelial ovarian cancer has been an interesting issue. However, previous studies in different laboratories provided highly controversial results (Kaufman *et al*, 1987; Leake *et al*, 1989; Beckmann *et al*, 1991; Lai *et al*, 1992,^1994^; Duggan *et al*, 1995; Runnebaum *et al*, 1995; Sworn *et al*, 1995; Trottier *et al*, 1995; Anwar *et al*, 1996; Kedzia *et al*, 1996; Mai *et al*, 1996; Zimna *et al*, 1997; Manolitsas *et al*, 1998; Anttila *et al*, 1999; Chen *et al*, 1999; Ip *et al*, 2002). We report here that HPV-16 was found in a series of histologically characterised epithelial ovarian carcinomas from Chinese women.

The oncogenic HPV product E6 targets p53 for degradation and therefore manipulates the host intracellular signal network in cervical cancers (Werness *et al*, 1990). Less study has been reported about p53 mutation and its relationship with HPV infection in ovarian cancer.

## MATERIALS AND METHODS

### Source and histological type of specimens

The patient group consisted of 50 epithelial ovarian carcinomas and four nonepithelial ovarian carcinomas. The mean age of the patients was 47 years (range 27–71 years). Histological types for 50 epithelial cancers were 24 serous cystadenocarcinomas, 19 mucinous cystadenocarcinomas, five endometrioid adenocarcinomas, and two undifferentiated carcinomas. Histological types for four nonepithelial cancers were two thecomas, one endodermal sinus tumour, and one malignant mesothelioma. The control group consisted of 30 pathologically confirmed nonmalignant ovarian tissues collected from 30 women (mean age 53 years; range 27–62 years). Among the nonmalignant tissues, 24 were ovaries removed for uterine pathology, five were ovarian cysts, and one was polycystic ovarian syndrome. Specimens were paraffin-embedded and archived from 1996–2000 in Inner Mongolia Medical College affiliated hospital.

Sections of 5 *μ*m thickness were cut from formalin-fixed, paraffin-embedded blocks. Tissue sections were attached on APES-treated glass slides for *in situ* hybridisation (ISH) and immunohistochemistry (IHC).

### *In situ* hybridisation

The full-length HPV-16 E6 gene was kindly provided by Dr zur Hausen. A 483 bp E6 probe was labelled with digoxin using an *in vitro* transcription kit (Boehringer Mannheim, Rocha Diagnostic GmbH, Agency Organisation GD-M Sandhoferstr-116, D-68305 Mannheim, Germany). Briefly, the *in vitro* transcription vector containing E6 gene was linearised by *SalI* digestion. The transcription was performed with T7 RNA polymerase in the presence of digoxin-labelled UTP and other unlabelled NTPs.

The paraffin sections were dewaxed, rehydrated and treated with 0.1 N HCl. The tissue slides were then digested with proteinase K at 37°C for 15 min. The HPV-16 E6 probe was mixed with hybridisation solution containing 50% formamide, 4 × SSC, 5% dextran sulphate, 5 × Denhardt's solution and 200 mg ml^−1^ ssDNA. The tissue slides were incubated overnight at 42°C in 20 *μ*l of the hybridisation solution with probe. The slides were washed in SSC. The E6 hybrid was detected by an alkaline phosphatase-conjugated antidigoxin antibody. The tissue slides were observed under a microscope. Pictures were taken for analyses. The HPV-16-positive oesophageal cancer was used as positive control (Li *et al*, 2001). The hybridisation solution without probes has been used as negative control. *In situ* hybridisation mostly detects the transcripts of a gene. The antisense probe hybridises with mRNA in the test system. Therefore, sense probe is a desired negative control. However, E6 integration in the host genome might result in transcription in both directions (Higgins *et al*, 1991; Vormwald-Dogan *et al*, 1992). Therefore, blank hybridisation was used as the control in this report.

### Immunohistochemistry

Specimens were dewaxed and treated with PBS containing 3% hydrogen dioxide. Slides were incubated at 37^0^C in normal goat serum for 15 min to eliminate nonspecific binding. Samples were incubated with either anti-HPV-16 E6 antibody (Santa Cruz Biotechnology, 2145 Delaware Ave., Santa Cruz, CA 95060, USA, 1 : 100 dilution) or anti-p53 antibody (Santa Cruz Biotechnology, 1 : 100 dilution) at 4°C overnight. The biotinylated secondary antibody and HRP-labelled streptavidin were then added and incubated at 37°C for 30 min. The signal was developed in DAB–H_2_O_2_ solution. The slides were counterstained with haemotoxylin.

### Statistical analysis

Statistical analysis was performed using *χ*^2^ test and odds ratio was used to determine prevalence of HPV-16 infection in epithelial ovarian cancers, nonepithelial ovarian cancers, and normal ovarian tissues.

## RESULTS

In 50 epithelial ovarian cancers, 26 were HPV-16 E6 positive determined by ISH. The positive rate is 52%. Only two or 6.7% of the normal ovarian tissue samples showed HPV-16 positive using ISH (Table 1

**Table 1:** HPV-16 E6 detection in epithelial ovarian carcinomas and normal ovaries by ISH

	**Tested case**	**Positive**	**Percentage**
Ovarian carcinoma	50	26	52
Normal ovary	30	2	6.7

*P*<0.01; *χ*^2^ test; OR=I6.7, 95% CI (3.2–71.4).

, *P*<0.01 OR=16.7 95% CI ∼3.2–71.4). Human papillomavirus-16 E6 was not detected in any of the nonepithelial ovarian cancers (Table 2

**Table 2:** HPV-16 E6 detection in different histological types of ovarian carcinoma

		**ISH**		**IHC**	
**Histological types**	***N***	***n***	**%**	***n***	**%**
Serous cystadenocarcinoma	24	15	62.5^a^	10	41.7^b^
Mucinous cystadenocarcinoma	19	8	47.4	5	26.3
Endometrioid adenocarcinoma	5	2		2	
Undifferentiated carcinoma	2	1		1	
Thecomas tumour^c^	2	0		0	
Endodermal tumour^c^	1	0		0	
Malignant mesothelioma^c^	1	0		0	

a *P*>0.05; *χ^2^* test;

b *P*>0.05; *χ^2^* test.

c Nonepithelial ovarian carcinomas.

). The purple signals represent HPV-16 E6 located mainly in the cytoplasm as described (Vormwald-Dogan *et al*, 1992) (Figure 1

**Figure 1:**
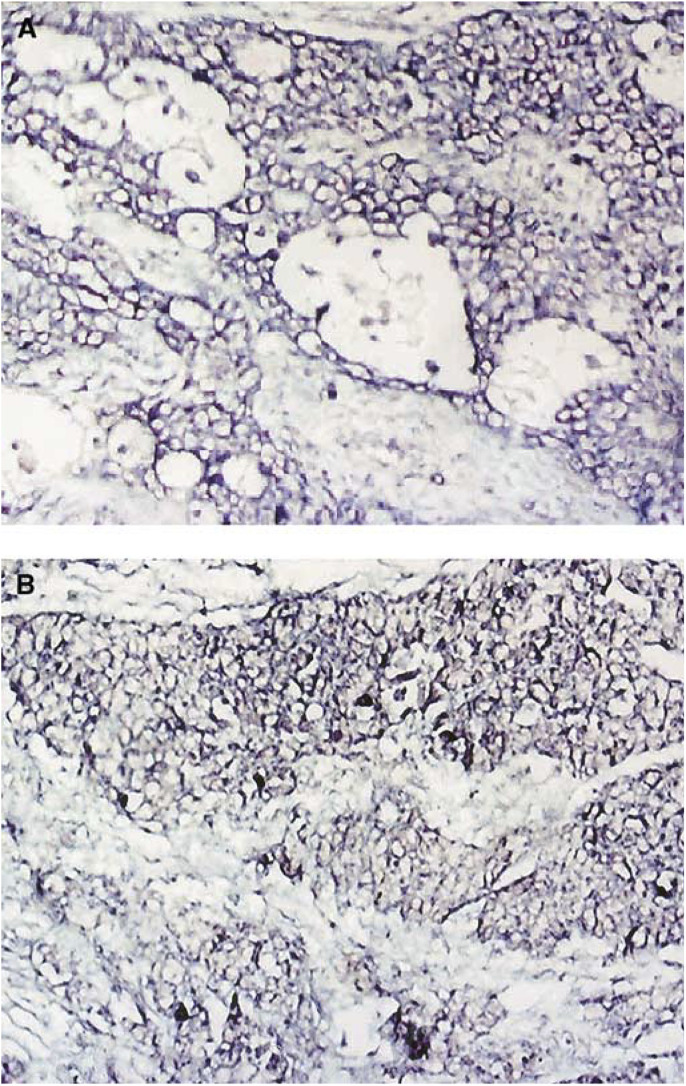
HPV-16 E6 in ovarian cancers by ISH, (**A**) sample no. 990917 and (**B**) sample no. 990193. The purple blue signals representing the expression of the E6 mRNA are at cytoplasm in the tumour cells. (**A**, **B**: original magnification × 100).

).

**Figure 2:**
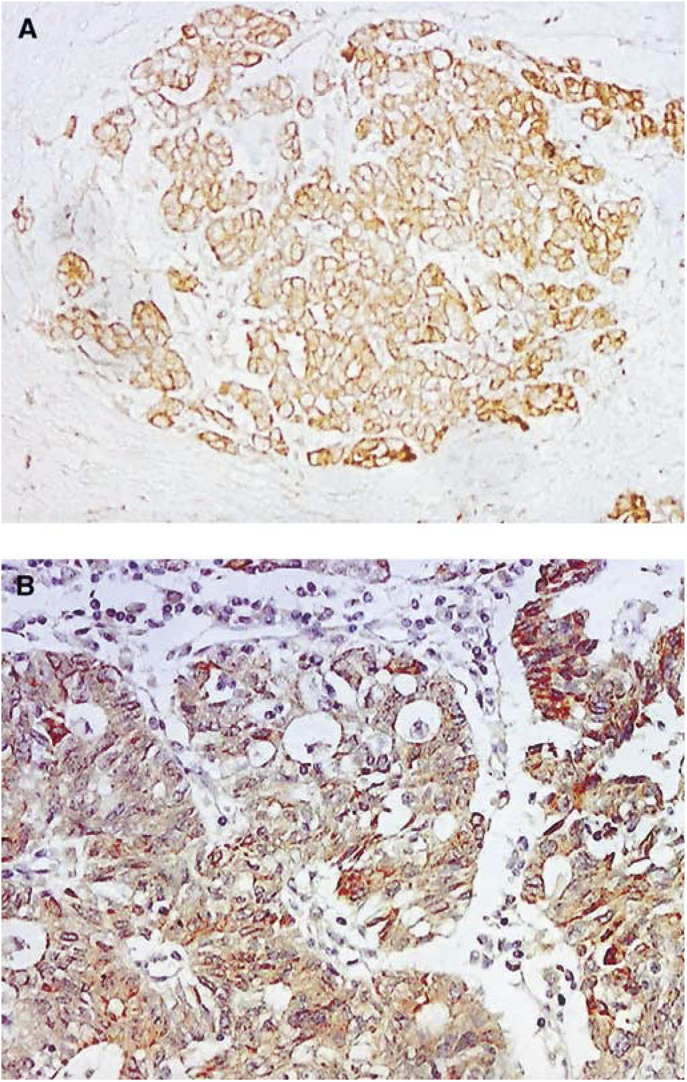
HPV-16 E6 in ovarian cancers by IHC, (**A**) sample No. 990917 and (**B**) sample no. 990589. The brown signals representing the expression of the E6 protein are mainly at cytoplasm in the tumour cells. (**A**, **B**: original magnification × 100).

Using immunohistochemical stain, 18 or 36% of the epithelial ovarian cancers were HPV-16 positive. All the positive samples detected by IHC were those positive samples detected by ISH. The brown signal located in the cytoplasm represents E6 protein (Figure 2

).

A total of 21 or 42% of the epithelial ovarian cancers were p53 positive. The brown signal located uniquely within the nucleus represents p53 protein. No correlation has been found between HPV-16 infection and p53 positive (Table 3

**Table 3:** Correlation between HPV-16 E6 infection and p53 mutation

	**HPV-16**	
p53	Positive	Negative
Positive	14	7
Negative	12	17

*P*>0.05; *χ^2^* test.

, *P*>0.05).

HPV-16 infection was not identified in any nonepithelial ovarian cancers, which are not the primary host for HPV. There was no correlation between HPV infection and histological types of ovarian cancers (Table 2, *P*>0.05). Owing to the sample size limitation, statistical comparison was only carried out between cystadenocarcinomas and mucinous cystadenocarcinomas.

## DISCUSSION

Human papillomaviruses play a causal role in cervical cancer. Human papillomavirus infection is also detected in other cancers of the female lower genital tract, including cancers of vulva, vagina, and perineum (Gupta *et al*, 1987; Shah *et al*, 1996; Dillner *et al*, 1997). However, the role of HPV infection in the development of cancers in the upper genital tract, such as endometrial cancer and ovarian cancer, is less clear. The first report on HPV infection in ovarian cancers was published in 1987 (Kaufman *et al*, 1987), although the article was retacted 1 year later by the authors. To date about 16 additional reports on HPV infection in ovarian tumours were found in the MEDLINE. Among these, 10 analysed more than 10 tumour specimens in each report (Leake *et al*, 1989; Beckmann *et al*, 1991; Lai *et al*, 1992,^1994^; Runnebaum *et al*, 1995; Trottier *et al*, 1995; Zimna *et al*, 1997; Anttila *et al*, 1999; Chen *et al*, 1999; Ip *et al*, 2002). Only in four of the 10 studies, HPV infection was found in ovarian cancers (Lai *et al*, 1992,^1994^; Zimna *et al*, 1997; Ip *et al*, 2002). It is interesting that in three of the positive reports, specimens of Chinese origin were used (Lai *et al*, 1992,^1994^; Ip *et al*, 2002). In the present study, a total of 54 ovarian cancers and 30 nontumour ovarian specimens were analysed for the presence of HPV-16. Among the 50 epithelial ovarian cancers, a total of 26 (52%) HPV-16 positive were found. Our results are consistent with those reports based on Chinese origin specimens. These independent studies are from three areas of grand China, Mainland China, Taiwan, and Hong Kong, suggesting that host genetic makeup may play an important role in susceptibility to HPV infection. Alternatively, HPV intratypic variants in different geographical regions may also determine the association with the risk of ovarian cancer. It has been reported that the distribution of HPV variants varied in different geographical areas, suggesting that the virus and the host have coevolved over time (Ho *et al*, 1993; Ong *et al*, 1993; Heinzel *et al*, 1995; Stewart *et al*, 1996; Yamada *et al*, 1995,^1997^; Hildesheim *et al*, 2002). Epidemiology studies in cervical cancers showed that individuals infected with a non-European variant of HPV-16 were associated with 2–9- fold increased risk of cervical cancer (Hildesheim *et al*, 2002). Whether this is the case in ovarian cancer needs to be further investigated.

In addition to the host and pathogen genetic variation, the difference of the detection methods employed in the studies might also account for data discrepancy. It has been well documented in cervical cancer that the episomal viral DNA frequently integrates into the host genome as HPV-infected lesions progress to cervical cancer. During viral DNA integration, only E6 and E7 genes remained (zur Hausen, 2002) in the host genome. Therefore, the presence of E6 or E7 genes in tumour tissues may better represent the real HPV infection. In some studies, L1 was used as the only indicator, which may have been lost or not expressed in the malignant specimens. We suggest that a well recognised standard for HPV detection in tumours should be issued for further studies.

In this study, we employed both ISH and IHC to detect HPV-16 E6 expression. Among the 26 HPV-16-positive cancer samples detected by ISH, 18 were confirmed positive by IHC. More importantly, none of ISH-negative samples were detected positive by IHC. These results indicate that IHC is an accurate method with less sensitivity.

The prevalence of HPV-16 infection in ovarian cancers is much higher than in nonmalignant ovarian tissues, suggesting that HVP-16 infection may play a role in the development of ovarian cancer. However, large case–control studies need to be conducted before reaching a conclusion.

Mutations of p53 are frequently detected in ovarian cancer (Crook *et al*, 1992; Kmet *et al*, 2003). Consistent with those studies, we found that 42% of ovarian cancers were p53 positive. The expression of p53 was not correlated with HPV infection. It has been proposed that the mechanism for the loss of p53 function in cervical cancer is due to degradation by HPV E6. Therefore, p53 mutation is rare in cervical cancers. However, there are reports suggesting that the association between HPV infection and p53 mutation differs among tumours (Hachisuga *et al*, 1996; Hasegawa *et al*, 2002), suggesting that additional mechanisms may be involved in tumorigenesis of other origins.
